# Thyroid Hormone and Wound Healing

**DOI:** 10.1155/2013/124538

**Published:** 2013-03-20

**Authors:** Joshua D. Safer

**Affiliations:** Section of Endocrinology, Boston University School of Medicine, Room M-1016, 715 Albany Street, Boston, MA 02118, USA

## Abstract

Although thyroid hormone is one of the most potent stimulators of growth and metabolic rate, the potential to use thyroid hormone to treat cutaneous pathology has never been subject to rigorous investigation. A number of investigators have demonstrated intriguing therapeutic potential for topical thyroid hormone. Topical T_3_ has accelerated wound healing and hair growth in rodents. Topical T_4_ has been used to treat xerosis in humans. It is clear that the use of thyroid hormone to treat cutaneous pathology may be of large consequence and merits further study. This is a review of the literature regarding thyroid hormone action on skin along with skin manifestations of thyroid disease. The paper is intended to provide a context for recent findings of direct thyroid hormone action on cutaneous cells *in vitro* and *in vivo* which may portend the use of thyroid hormone to promote wound healing.

## 1. Introduction

Despite early observation of cutaneous pathology associated with thyroid disease [1, 2], research on the topic remains sparse. Direct thyroid hormone action has been demonstrated on cutaneous biology including on the epidermis, dermis, and hair. In addition, autoimmune diseases with cutaneous manifestations may be associated with thyroid dysfunction (which may be autoimmune in etiology itself).

The lack of investigation into cutaneous responses to thyroid hormone might be attributed to the fact that mostcases of thyroid disease are controlled with existing medication. However, there is a slowly evolving literature that suggests that the thyroid hormone pathway is integral to cutaneous physiology and that manipulation of the thyroid hormone pathway in skin could be used to treat cutaneous disease. 

The skin manifestations of hypothyroidism have been described since the 1800s. Indeed, the observation associating hypothyroidism with the skin [1] predates the classic publication associating hypothyroidism with the thyroid [2].

Thyrotoxicosis is also associated with cutaneous manifestations [3]. Although autoimmune-associated manifestations may be specific to Graves' disease, thyrotoxicosis in general may result in skin sequelae. Starting in the 1950s, there were attempts to use parenteral and topical tri-iodothyronine (T_3_) to treat pretibial myxedema in Graves' patients [4–7]. In all cases, lesions improved with topical or intralesional steroids which ended further study of thyroid hormone action. At that time, it was noted that topical thyroxine (T_4_) stimulated hair growth and pigmentation in cows [8]. 

## 2. Direct Thyroid Hormone Action on Skin Tissues

 Thyroid hormone action on skin is mediated through the thyroid hormone receptor (TR). The three most recognized thyroid hormone binding isoforms of TR have been found in cutaneous tissues [9–12] although methods used do not specifically distinguish which of the three isoforms predominates.

 The predominant circulating thyroid hormone is the prohormone, T_4_. T_4_ is converted to the active thyroid hormone, T_3_, by intracellular thyroid hormone deiodinases [13, 14]. Two of the enzymes (D1, D2) primarily activate T_4_ to T_3_. The third enzyme, D3, converts T_4_ to inactive reverse T_3_ (rT_3_). Investigators have showed conversion of T_4_ to either T_3_ or rT_3_ in cutaneous cultures, demonstrating indirectly the presence of thyroid hormone deiodinases in skin [15–17]. D3 is not expressed significantly in most peripheral tissues. However, assays of enzyme activity suggest that D3 is active in goat epidermis [18], mouse epidermis [19], and human skin *in vivo* [20, 21]. D2 activity has been demonstrated in cultured human fibroblasts [22]. Neither D1 nor D3 has been found to be active in the dermal fibroblasts, suggesting that D3 expression may be limited to epidermis.

 In addition, investigators have identified elements of the hypothalamic-pituitary-thyroid hormone axis in human skin [23–25] and have determined that thyroid hormone receptors mediate skin proliferation and inflammation along with skin response to retinoids [26, 27].

### 2.1. Epidermal Changes

Thyroid hormone accelerates barrier formation by increasing the activity of enzymes in the cholesterol sulfate cycle, and hypothyroidism may hinder the epidermal barrier function [28, 29]. Hypothyroidism also may affect the development of the lamellar granules (Odland bodies), which are vital in the establishment of a normal stratum corneum [30]. *In vitro* keratinocyte studies have shown that depletion of T_3_ results in elevated levels of transglutaminase (involved in the formation of the cornified envelope). Further *in vitro* analysis has suggested that T_3_ depleted keratinocytes have diminished levels of plasminogen activator [31].

In tissue culture studies, T_3_ has been shown to directly stimulate proliferation of both epidermal keratinocytes and dermal fibroblasts [32–34]. However, *in vivo*, skin proliferation directly stimulated by T_3_ may be offset by inhibiting factors dependent on the systemic T_3_ [33]. Clinical observations of skin in thyrotoxic patients are complicated by the fact that most cases of thyrotoxicosis are the result of Graves' disease which has an associated finding of autoimmune-mediated glycosaminoglycan deposition in the dermis [35]. 

### 2.2. Dermal Changes

The skin tends to be pale in hypothyroidism both because of the dermal mucopolysaccharides and the dermal water content. Changes in hypothyroid dermis can be seen in weeks and reversed in weeks [36, 37]. Hyaluronic acid is the major glycosaminoglycan that accumulates in myxedema [38]. In addition, lack of lymphatic drainage may explain the formation of exudates that are apparent in the myxedematous state [39]. Increased dermal carotene may appear as a yellow hue on the palms, soles, and nasolabial folds. *In vitro*, thyroid hormone actions on cultured skin fibroblasts include inhibiting synthesis of hyaluronic acid, fibronectin, and collagen [40–42].

The net effect of thyroid hormone on dermal thickness remains the subject of debate. In the past, investigators have reported skin thinning in rats made thyrotoxic with intraperitoneal (IP) T_4_ [43]. There have been demonstrations both of decreased collagen production in the thyrotoxic animals and increased collagen catabolism in thyrotoxic animals [43, 44]. More recent investigations suggest increased dermal thickness in mice treated with T_3_, whether topically or intraperitoneally [32, 33]. There is also a report of increased dermal thickness in mice treated topically with TRIAC (a thyroid hormone analog [45]).

### 2.3. Hair and Nail Changes

Hale and Ebling demonstrated that intraperitoneal T_4_ decreased both the resting phase of the hair growth cycle (telogen) and the growth phase of the hair growth cycle (anagen). Although there was an enhanced turnover, the net hair length at any given time was not changed from that of untreated animals [46]. The time to regrowth of hair following epilation was shortened by approximately 10%. 

In hypothyroidism, hair can be dry, coarse, brittle, and slow growing. Nails may be thickened, brittle, and slow growing [47]. Diffuse or partial alopecia may be observed along with loss of the lateral third of the eyebrow (madarosis). The alopecia connected to hypothyroidism may be mediated by hormone effects on the initiation as well as the duration of hair growth. There is one report of long, terminal hairs on the backs and extremities of hypothyroid children [48]. The hair disappeared following thyroid hormone replacement, but no mechanism was determined.

Clinically, the hair in thyrotoxicosis may be fine and soft. Nail changes may also occur, characterized by a concave contour and distal onycholysis (Plummer's nails). Diffuse, nonscarring alopecia may also be observed.

Like with epidermal proliferation, hair changes with thyrotoxicosis are different than what might be seen with topically administered thyroid hormone. Mice and rats treated daily for 1-2 weeks with topical T_3_ had increased hair counts, but mice made thyrotoxic with daily intraperitoneal T_3_ for 1-2 weeks had decreased hair counts [33, 44]. Thyrotoxic goats had increased mohair length but decreased fiber diameter [49]. A topical mixture including thyroxine, insulin, and growth hormone increased hair counts over a 6-month treatment period in men with androgenic alopecia [50].

The dryness of hypothyroid skin results from decreased eccrine gland secretion. The mechanism for decreased sweating is not clear although the glands are atrophic on histologic examination [51]. Hypothyroidism has been reported to be a cause of increased sweat electrolytes, requiring differentiation from cystic fibrosis [52].

Hypothyroid patients may sometimes suffer *Candida *folliculitis. It has been theorized that this may result from decrease sebum production relative to euthyroid persons. The hair follicles may develop a flora with fewer lipophilic organisms, which are replaced by *Candida albicans* [53].

## 3. Potential Use of Thyroid Hormone in the Treatment of Cutaneous Pathology

Knowledge of cutaneous manifestations of thyrotoxicosis does not predict the direct effects of T_3_ on skin *in vivo*. T_3_ effects on skin depend on route of delivery. Because epidermis expresses the inactivating type 3 deiodinase [19], thyroid hormone action on epidermis in systemic thyrotoxicosis is less than might be predicted. In contrast to the findings with systemic thyrotoxicosis, topical T_3_ bypasses the inactivating type 3 deiodinase and stimulates epidermal proliferation, dermal thickening, and hair growth [32, 33]. Topical application of TRIAC (tri-iodothyroacetic acid), the thyroid hormone analog, thickens skin by stimulating production of collagen [45]. Further, topical TRIAC has been shown to reverse the dermal atrophy associated with corticosteroids.

The importance of thyroid hormone in wound healing had been debated historically. In 1973 and 1974, Mehregan and Zamick reported that oral T_3_ accelerated the rate of wound healing in euthyroid rats and improved the quality of the wounds [54, 55]. Scars were smoother in T_3_-treated animals. Lennox and Johnston reported accelerated wound healing and increased tensile strength when rats were given supraphysiologic doses of T_4_ [56]. Pirk et al. reported no change in wound healing with 1.3 *μ*g/100 mg body weight intraperitoneal T_4_ in hamsters but increased rate of fracture repair [57]. Ashton and Dekel also reported increased fracture repair rate in mice given 20 *μ*g/100 mg body weight subcutaneous T_4_ [58]. In small series, there are reports that hypothyroid patients required thyroid hormone to achieve healing of radiation-induced neck fistulae [59, 60]. Conversely, Cannon [61] reported that hypothyroidism did not diminish wound strength in pigs, and Ladenson et al. [62] did not detect wound healing deficits in hypothyroid humans.

In contrast, most recent data suggest that topical thyroid hormone may accelerate wound healing rate. Topical application of supraphysiological doses of T_3_ accelerated wound healing in *normal* mice and rats [63–65]. A human wound healing formulation has been described that requires T_4_ in addition to growth hormone and insulin [66]. 

While direct thyroid hormone action has been demonstrated on cutaneous cell biology, much more study remains to be done. There is a slowly evolving literature to suggest an important role for thyroid hormone in cutaneous wound repair that could be harnessed as a pharmaceutical.
